# Do Longer Fins Improve Ocean Rescues? A Comprehensive Investigation into Lifeguard Performance and Physiological Impact

**DOI:** 10.3390/jfmk9020079

**Published:** 2024-04-19

**Authors:** Isaac Ignacio-Rodríguez, Roberto Barcala-Furelos, Ezequiel Rey, Marcos Sanmartín-Montes

**Affiliations:** REMOSS Research Group, Faculty of Education and Sport Sciences, Universidade de Vigo, 36005 Pontevedra, Spain; isaacrodriguezignacio@gmail.com (I.I.-R.); zequirey@uvigo.es (E.R.); marcos.sanmartin@uvigo.es (M.S.-M.)

**Keywords:** lifesaving, fins, water rescue, lactate, effort, lifeguards, drowning

## Abstract

Coastal environments present dynamic challenges necessitating rapid and efficient responses during aquatic emergencies. Lifeguards, as pivotal links in the intervention chain, rely on various tools, with rescue time being paramount. The choice of fins, specifically long versus short ones, plays a critical role in optimizing lifeguard performance during rescues. This randomized cross-over study explores the impact of flipper size on ocean rescues, employing a sample of 14 lifeguards. Long fins (LFs) and short fins (SFs) were compared in terms of rescue time (RT) and physiological load (PL). Tests included ocean rescues without fins (R), with LF (R-LF), and with SF (R-SF). Variables recorded encompassed swim approach time, tow-in time, overall rescue time, perceived exertion rates (RPEs), and post-rescue lactate concentration. Long fins demonstrated superior performance in swim approach and tow-in times compared to both short fins and no fins (*p* < 0.001). Overall rescue time favored long fins significantly (*p* < 0.001), indicating their efficiency in practical ocean rescue scenarios. Physiologically, long fins induced lower perceived exertion in arms (*p* = 0.033) compared to short fins. Lactate concentrations post-rescue revealed no significant differences (*p* > 0.05). This study demonstrates that long fins significantly improve lifeguard performance during ocean rescues, reducing rescue times and alleviating arm fatigue.

## 1. Introduction

These coastal environments pose unique and dynamic challenges that demand a swift and effective response in emergency situations. The coast exhibits significant variations across its geography, ranging from beaches situated in bays or estuaries with calm waters to those located in areas with strong currents and big waves. Every year, countless lives are saved thanks to lifeguards performing prevention, surveillance, and intervention functions on the beaches [1]. When establishing a response chain to an aquatic incident [2], achieving the fastest possible rescue is crucial to stop the drowning process, as submersion time is a strong predictor of survival [3]; hence, the lifeguard’s actions emerge as the key link in aquatic intervention [4].

Lifeguards play a crucial role in preventing drowning, which is defined as “the process of experiencing respiratory impairment from submersion/immersion in liquid” [5]. Drowning accounts for almost 300,000 deaths annually, ranking among the top 3 causes of death in numerous countries. However, this fatal outcome can be mitigated by the timely interventions of rescuers in the water. In cases of drowning, it is essential not only to ensure the survival of the victim but also to minimize the associated damage. This damage can range from death to neurological impairment, with the quantity of water ingested and the duration of submersion being critical factors [3,6]. Thus, swift intervention to halt submersion is a primary objective. Equally important is the prompt return to land to administer necessary care and reduce the risk to the rescuer.

In order to execute rescues effectively and safely, lifeguards rely on various materials to enhance their propulsion or buoyancy [7]. One fundamental element for lifeguards is the use of rescue fins [8], since many of the ocean rescues will take place between 50 and 100 m from the coast [4,9]. This type of equipment enables the rescuer to reach the victim more quickly, thereby reducing the risk of complications from drowning and increasing the chances of survival [10]. Studies have demonstrated the correlation between the duration of submersion and the severity of consequences [3,6], underscoring the critical importance of prompt rescue. However, it is imperative not to overlook the safety of the lifeguard. Most rescues involve swimming and “melee” techniques [10], which can pose risks to the rescuer’s safety due to the victim’s anxiety and nervousness [11]. The use of fins enables the rescuer to manage such scenarios more efficiently and under safer conditions for both themselves and the victim [12].

In the lifeguard scientific literature, numerous studies have analyzed the influence of fins, both in the pool [13,14,15,16,17] and in natural environments such as beaches [7,8]. However, there are still numerous areas where our understanding is incomplete, such as the performance of rescues in coastal environments, depending on the size and type of fins.

In the realm of lifeguards, two main types of fins are commonly encountered: a short model (body board) or a longer one (for swimming). The choice of material is typically influenced by local customs or personal preferences [7]. Numerous studies have demonstrated how water rescues performed with this equipment are more effective in terms of reduced time spent or the distance covered by the lifeguard [7,8,14,15,16,17]. Faster rescues not only entail less exposure to risk or water aspiration [7,18] but also allow for early initiation of CPR, potentially increasing the survival chances of a drowning victim or minimizing neurological damage [3,19]. Additionally, the use of fins enables lifeguards with weaker swimming skills to optimize their performance, reaching speeds comparable to the best swimmers [14].

From a hydrodynamic perspective, fins offer greater propulsion by improving the force fraction that is useful for propelling the body forward and mechanical efficiency [20]. There are various models of fins available to lifeguards, each with different lengths and made from different materials [8,13,14,15]. Fins with a longer blade provide better propulsion conversely, shorter fins favor a higher kick frequency [21,22] and lower energy expenditure [23].

If we specifically consider swimming time and the time required for the rescuer to equip themselves with fins, it has been suggested that shorter fins are more suitable for rescues at a distance of 50 m from shore, while longer fins are preferable for rescue situations at 100 m [16]. However, during rescues, in addition to the time taken to reach the victim, we must also take into account the time required for towing them back to the coast [4,16,24]. Considering this aspect, the use of fins results in less decrease in speed when analyzing towing in swimming pools compared to not using them [13,14,15]. Delving further into which type of fin is more effective for towing the victim in the pool, there is a consensus that longer and stiffer fins generate higher levels of speed and experience lower descents [14,15,17]. The truth is that aquatic rescue is a strenuous and physiologically demanding activity [25], alleviated by using fins in terms of lower energy cost [8]. However, the evidence is controversial, as some studies argue for lower energy costs with shorter fins [21], while others show a lower fatigue index when using longer and rigid fins [15,17]. However, the majority of these studies have been conducted in pools. The reality is that lifeguards regularly use fins in ocean rescues, creating a knowledge gap that focuses research and findings on a more realistic environment. To our knowledge, there are no studies in natural aquatic environments (beaches, lakes, rivers, etc.) comparing rescue times and physiological responses based on fin size.

Our hypothesis is that longer fins may offer better performance to lifeguards, especially when they have to tow-in a victim to shore. Therefore, the objective of this study is to compare rescue time and the lifeguard’s physiological response based on the fin model (long or short).

## 2. Materials and Methods

### 2.1. Study Design

**Figure 1:**
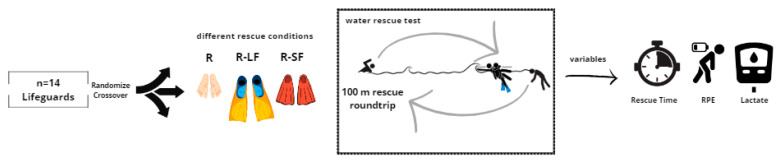
Flow chart design. R, rescue without fins; R-LF, rescue with long fins; R-SF, rescue with short fins; RPE, rate of perceived exertion.

A randomized cross-over quasi-experimental study design was used to analyze the differences between ocean rescues using long fins (LFs) versus short fins (SFs) in terms of rescue time (RT) and physiological load (PL) (Figure 1).

### 2.2. Sample

A convenience sample of 14 lifeguards (4 women and 10 men) volunteered to participate in this study. Participant characteristics were as follows: age 22.5 ± 5.1 years, height (cm) 181 ± 1.7, weight (kg) 75.71 ± 12.2, and body mass index 24.74 ± 3.5. The lifeguards underwent training at the Faculty of Education and Sports Sciences of the University of Vigo (in the city of Pontevedra, Spain) and received rescue swimming training twice a week for the past 4 months (September to December). This training and education focused on technical aspects (rescue with and without equipment and first aid skills) and physical conditioning relevant to lifeguarding, qualifying them for the tasks of a professional lifeguard. In addition to lifeguard training, participants receive complementary training in other subjects of the Sports Science degree, such as swimming, rowing, or athletics, which may have direct transferability to rescue skills, knowledge of the environment, and optimal physical fitness.

Inclusion criteria for the study were as follows: having knowledge and experience in lifeguarding, possessing experience in swimming with both long and short fins, and not suffering from any disabling condition for participating in the study. Furthermore, they must have training for lifeguard development according to local legislation (professional certification or the contents passed with any legal training for obtaining the professional card from the Galician Lifeguard Registry, Spain). All participants provided signed informed consent. This study received approval from the Ethics Committee of the Faculty of Education and Sport Sciences at the University of Vigo (Spain) with the code 11-181223.

### 2.3. Characteristics of Equipment

**Figure 2:**
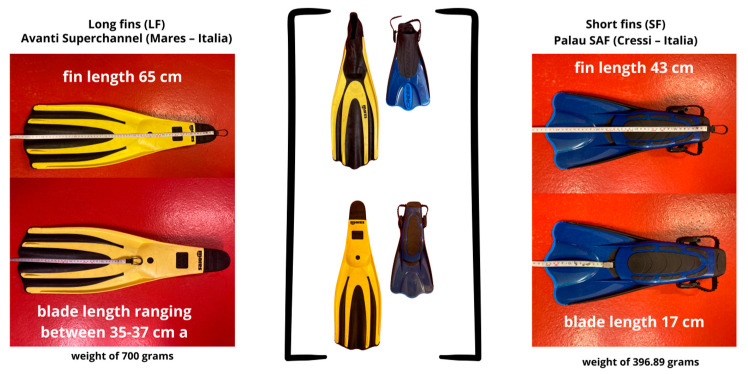
Fins characteristics.

Two models of swim fins available in the market were utilized. The long swim fins used were the Avanti Superchannel model (Mares, Rapallo, Genoa, Italy) with a blade length ranging between 35 and 37 cm with a weight of 700 g, and the short swim fins were the Palau SAF model (Cressi, Genoa, Italy) with a blade length 15 cm, with a weight of 396.89 g. For victim rescue and towing, a rescue tube of the MARPA model (Emergalia, San Cibrao das Viñas, Ourense, Spain) was employed, with dimensions of 100 cm × 16 cm × 9 cm. All lifeguards conducted the test wearing their personal wetsuits, with a thickness of 3.2 mm (Figure 2). According to International Swimming Federation (FINA) regulations for open water competitions, the use of wetsuits is mandatory for temperatures below 18 °C [26]. In this study, we have followed this recommendation for the safety and prevention of hypothermia for the lifeguards.

### 2.4. Trial Characteristics (Figure 3)

Three tests were conducted: (1) ocean rescue without fins (R), (2) ocean rescue with long fins (R-LF), and (3) ocean rescue with short fins (R-SF). Lifeguards started with water at waist level. Upon the instructor’s signal, they began the test by donning the fins (except in the no-fins test) and then swam towards the victim, who was positioned face down at a distance of 100 m from the shore, simulating unconsciousness. The objective was to tow-in the victim to land as quickly as possible. The victim, dressed in a wetsuit, simulated unconsciousness, floating face down and facing away from the shore at the time of contact. The anthropometric characteristics of the victim were similar to those of the rescuer, with a deviation of no more than 5 kg in weight and 10 cm in height. The victim remained the same for each lifeguard during all three tests.

To mitigate bias from sea-state variability, the tests were performed in rounds of three participants (one without fins, another with long fins, and another with short fins). To avoid fatigue bias, a one-hour break was established for each participant between each test. To eliminate bias from meteorological conditions, all tests were conducted on the same day within the time frame of 12 to 4 p.m.

### 2.5. Variables and Measuring Procedures

Two sets of variables were recorded:

1. Rescue Time: Expressed in seconds, broken down into (a) time to approach the victim, (b) time to tow the victim, and (c) total rescue time. This variable was recorded by two members of the research team using the stopwatch app on the iPhone SE with iOS 17. To discriminate between the approach time and tow-in time, the victim is instructed to raise their right hand as a signal that the lifeguard begins tow-in (second phase) when the rescuer initiates the first pull towards the shore.
2. Physiological Variables: (d) Rate of perceived exertion (RPE): Measured on an index from 0 to 10 according to Foster’s scale, RPE ranges from 0 meaning “totally rest” to 10 meaning “maximal effort” [27], disaggregated into four values (overall RPE, chest, arms, and legs); (e) Blood lactate concentration post-rescue: For lactate measurements, the Lactate Scout device (SensLab GmbH, Leipzig, Germany) was used, and the results were expressed in mmol/L. Lactate measurements were taken within the first minute after the test.

### 2.6. Weather Conditions

The trial took place on Mogor Beach in the province of Pontevedra, located in the northwest part of Spain. In the northwest of Spain lies the region of Galicia, surrounded on three-quarters of its territory by the sea. Its southern area, Pontevedra, is bathed by the waters of the Atlantic Ocean, with average annual water temperatures of 14 °C, rising to 18 °C in summer. Wave heights vary from calm seas to rough seas according to the Douglas scale. The average annual ambient temperature is 16 °C. Mogor Beach, shaped like a bay, is typically sheltered from the wind and characterized by mild currents and waves.

The average weather conditions during the trials were as follows: water temperature: 14 °C, average air temperature: 12 °C, sea state: 0 on the Douglas scale (calm sea), and wind speed: less than 5 knots. These data were obtained from the local meteorological agency, www.meteogalicia.gal accessed on 19 December 2023.

### 2.7. Statistical Analysis

Data are presented as means and standard deviation (SD). All statistical analyses were conducted using the statistical package SPSS for Macintosh (version 25.0, Chicago, IL, USA). A 3 (R vs. R-SF vs. R-SF) repeated-measures analysis of variance (ANOVA) was calculated for each variable. Partial eta squared (ηp^2^) effect sizes (ES) were calculated. An effect of ηp2 ≥ 0.01 indicates a small, ≥0.059 a medium, and ≥0.138 a large effect [28]. When appropriate, pair-wise comparisons were conducted using the Bonferroni post hoc test. Additionally, Cohen’s *d* was computed for comparing effect sizes for pair-wise comparisons. Cohen’s *d* values were classified as trivial (*d* < 0.2), small (0.2 ≤ *d* < 0.5), moderate (0.5 ≤ *d* < 0.8), and large (≥0.8) [28].

## 3. Results

The results presented are based on a total of 42 rescues (three per lifeguard), at a distance of 100 m from the shore to a victim simulating unconsciousness and under calm sea conditions.

The differences in rescue time variables between the R, R-LF, and R-SF are shown in Table 1.

For the variable “swim to approach”, statistically significant results were found, with the time taken using long fins being less than that without fins (*d* = 0.82, large) or with short fins (*d* = 1.42, large). No significant differences were found between the approach time using short fins and the approach time without fins (d = 0.06, trivial).

For the variables “tow-in to the shore” and “overall rescue time”, significant differences were found in all comparisons, with the most favorable result obtained with long fins, followed by short fins (+55 s difference with R-LF), and finally, the rescue without fins (+90 s difference with R-LF).

**Figure 4:**
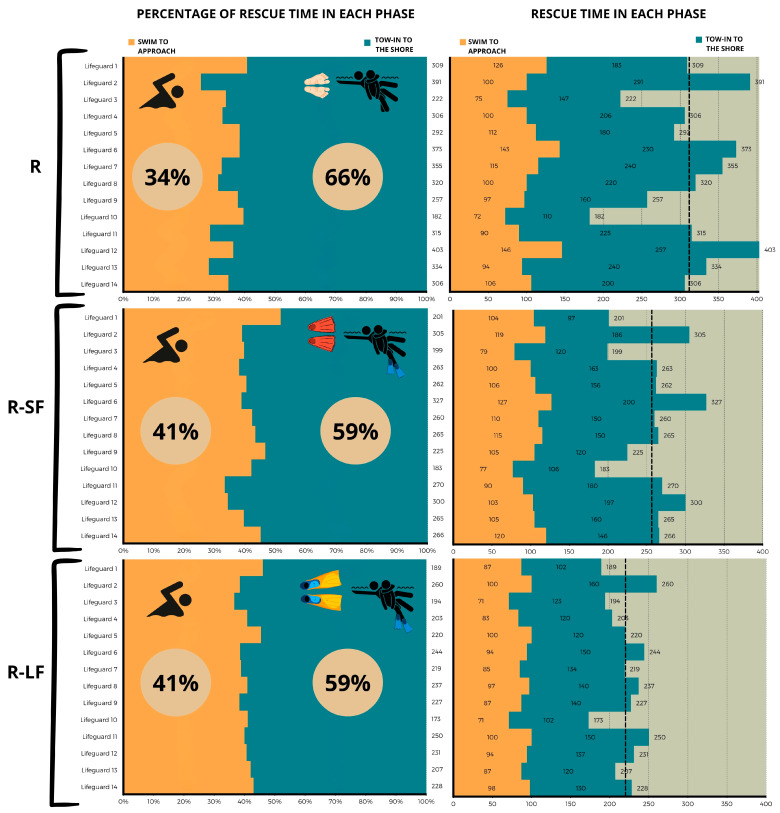
Rescue time variables.

The percentage distribution of time for each phase was as follows: for R, swim to approach was 34%, and tow-in to the shore was 66%. For R-SF, swim to approach was 41%, and tow-in to the shore was 59%. Finally, for R-LF, swim to approach was 41%, and tow-in to the shore was 59% (Figure 4). Analyzing the 7 fastest lifeguards versus the 7 slowest, the former had an average advantage of 88 s over the latter when not using fins, 57 s with short fins, and 30 s with long fins.

The analysis of physiological variables is presented in Table 2.

In the RPE analysis, it was found that the use of long fins (*d* = 0.98, large) and short fins (*d* = 0.96, large) resulted in lower perceived exertion in the arms. Additionally, it was observed that the use of long fins led to lower overall RPE compared to rescuing without fins (*d* = 0.67, moderate). No statistically significant differences were found in the perceived exertion in other evaluated areas (legs and chest) (*p* > 0.05). The blood lactate concentration was higher than 7 mmol/L, with no statistically significant differences among the three rescue conditions (R, R-SF, and R-LF) (*p* > 0.05).

## 4. Discussion

The objective of this study was to compare rescue time and the lifeguard’s physiological response based on fin model (long or short), and the main findings were as follows: (a) Both fin models substantially improve the total rescue time; however, long fins offer superior performance in all phases of the rescue (swim to approach and tow-in to the shore) compared to short fins, and (b) an aquatic rescue, whether with or without fins, generates a significant physiological demand with high concentrations of lactic acid and elevated perceived exertion (RPE); nevertheless, long fins manage to attenuate perceived fatigue in the arms.

Lifeguards employ various auxiliary devices for lifesaving [7], but fins hold particular significance, reducing rescue time and enhancing aquatic maneuverability in rip currents or waves [8]. Consistent with prior research [7,8,16], our study demonstrated that the use of fins, especially the long model, significantly improved aquatic rescue time.

In this context, what does our study contribute to the knowledge gap? Typically, rescue analyses focus on the end result, primarily evaluating how quickly a drowned person is brought to shore. However, in the analysis, the time taken to reach the victim should also be considered relevant. The most crucial prognostic factor for a favorable neurological outcome is the shortest submersion time [3]. Therefore, reaching the victim as quickly as possible and interrupting the drowning process is a fundamental aim.

In our study, when the lifeguards must cover a certain distance (100 m swim to approach), long fins provide an advantage over short fins. However, short fins do not confer a significant advantage compared to swimming to approach without fins. If we specifically focus on swim time and the time required for the lifeguard to don the fins, it has been suggested that shorter fins are more suitable for rescues at a 50 m distance from the shore, while longer fins are preferable for 100 m rescue situations [16]. This may be related to the characteristics of the approach swim, where the lifeguard needs to raise their head to observe the trajectory. Hydrodynamics are worse when facing waves or currents, and long fins offer more traction, overcoming marine environment resistances more effectively [8].

Another noteworthy aspect is the 7% reduction in the tow-in to the beach phase when fins are used, translating to a time saving of 54 s for R-SF and 76 s in R-LF, minimizing the risk to the lifeguard and the risk of water aspiration for the victim. Thus, the results shown are aligned with statistical data showing how drowning rescues last 5 min or less [10] and how those with high probabilities of survival do not use more than 10 min [6].

In the effort analysis carried out by the lifeguards during the rescue, it falls within a range above 6 for RPE and above 7 mm/L for lactate. These data represent a medium–high intensity, but not an extreme one, possibly because lifeguards regulate their aquatic rescues at a “safety” intensity that allows them to reach the shore and be able to extract and continue care. While lactate results do not show significant differences, it is interesting to note that efforts are above the considered anaerobic threshold, indicating a type of lactate anaerobic effort [29,30]. In this study, the mean lactate values reached were close to or lower than those obtained in other studies with lifeguards that conducted various trials comparing materials or rescue conditions [7,8,31,32]. The differences may be due to the fact that in the present study, all the rescuers used wetsuits, which helped them to reduce resistance to advance and achieve a lower lactate accumulation [33,34].

As for the RPE variable, a significantly lower value was found in the arms when long fins were used. This can be explained by the rescue technique when fins are not used, and towing actively engages the upper limbs, with one arm performing propulsive strokes. However, why is there also a significant difference in arm fatigue between rescues with fins? It is possible that arms may play a more relevant role in tow-in with short fins, providing support at various moments (e.g., changing trajectory or using auxiliary strokes to maintain the hydrodynamic position).

This study involved 14 lifeguards, each with a wide range of aquatic skills. It was observed that the use of fins helped reduce the time differences between those categorized as “slower swimmers” compared to faster swimmers when not using fins. The slower swimmers were nearly 90 s slower than the faster swimmers when not using fins; however, with long fins, they were only 30 s slower. These results suggest that lifeguards with poorer swimming skills should prioritize the use of fins, especially in long-distance rescues.

Essentially, fins serve as a tool for rescue teams to balance their varying skills and competencies, fostering more balanced and efficient teams. Rescue trials with lifesaving manikins in swimming pools concluded that fins enable lifeguards with less proficiency in swimming to achieve rescue times comparable to those of proficient swimmers [15].

### 4.1. Practical Implications

The practical implications derived from the study findings are significant for lifeguard teams and water rescue operations. Here are some key practical implications:

Material selection for rescue: The study provides valuable insights into choosing the most suitable equipment for water rescue operations based on local conditions and the nature of aquatic incidents. Lifeguard teams can use this information to make informed decisions on whether to use long or short fins, considering factors such as swim distance and environmental conditions.

Training and physical preparation: The study offers guidance for training programs and physical preparation tailored to the demands of rescue efforts lasting approximately 3–4 min at intensities close to the lifeguard’s maximum oxygen consumption (VO_2_max) [31,32,35]. In addition, the provided data demonstrate how specific training in the crawl kick is crucial for undertaking a rescue with fins under optimal conditions. Therefore, a training program for this rescue group should focus on efforts close to VO_2_max and enhancing propulsion of both the upper and lower limbs. Lastly, considering the variability of conditions in which the lifeguard must perform their duties, a high level of technical skill is necessary.

Performance consistency: Lifeguards with varying swimming skills can benefit from the use of fins, especially long ones, to minimize performance differences. This is particularly relevant when facing rescues requiring swimming distances between 100 and 200 m.

Equipment selection for specific scenarios: The study suggests that long fins may be a preferred choice for longer swim distances, while short fins are ideal for use in inflatable rescue boats or rescue watercrafts due to their compact size and minimal distance on swimming to the victim.

Simulation, research, and implementation in new scenarios: In this study, a typical rescue was analyzed in which long fins performed better. To provide a broader selection criterion for fins, tests should be implemented at other distances, with waves, or in situations involving multiple rescues.

### 4.2. Study Limitations

This study has faced several limitations that need to be considered. Firstly, the sample size was small and convenience-based, and the tests were conducted in ideal weather conditions. It is crucial to acknowledge that results could vary under different conditions. This is a simulation study, so performance in a real rescue in terms of motivation, hormones, and physiology is very different from a training situation.

The tests were conducted in groups, so that two or three lifeguards could perform the test simultaneously, and occasionally, there might be some motivational interference (e.g., competitiveness). However, an adequate separation was established, and the rescuers were instructed to behave as they would in a real rescue.

The lifeguards wore wetsuits as a preventive measure against hypothermia due to the water being at 14 °C. The use of wetsuits could alter rescue times and overall physiological parameters compared to not using them. Another fundamental limitation was that the victims were simulated by other rescuers dressed in wetsuits, so the difficulty of the test, hydrodynamics, fatigue, and rescue time could vary when rescuing a victim in a real context. In such cases, a victim without a wetsuit, in an agitated or unconscious state, would increase resistance to advancement. Additionally, all first responders and victims had a BMI below 25. Future research should address scenarios involving overweight or obese victims, situations of prior fatigue (e.g., running through the sand to the water), or complete extraction to dry sand and the continuity of care (ABCD approach). Under these conditions, the results could vary.

## 5. Conclusions

This study demonstrates that long fins significantly improve lifeguard performance in ocean rescues, reducing rescue times and alleviating arm fatigue. The findings emphasize the importance of flipper selection, highlighting the practical benefits of long fins over short ones in real-world rescue scenarios. Additionally, the use of fins helps lifeguards with less developed aquatic skills achieve rescue times closer to those of faster lifeguards, a crucial factor when forming a rescue team given the heterogeneity among rescuers. Furthermore, the effort faced by a lifeguard in a water rescue is of a lactic anaerobic nature, where the legs play an exceptionally important role in propulsion both with and without fins. These aspects should be taken into account when designing training programs for rescue teams.

## Figures and Tables

**Figure 3:**
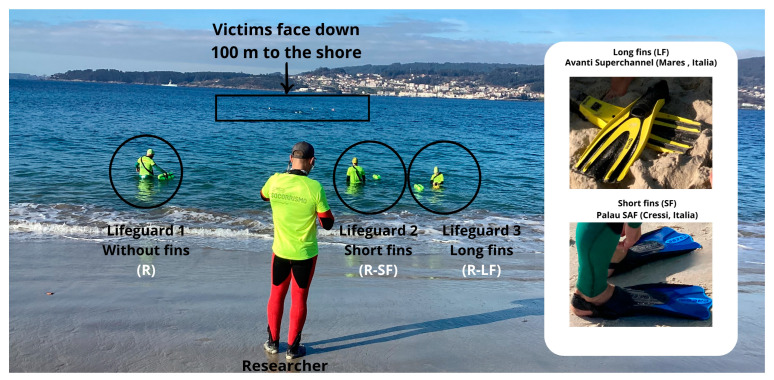
Trial representation and fins characteristics.

**Table 1:** Rescue time variables.

Ocean Rescue Variables	R	R-SF	R-LF	*p*-Value	Bonferroni Test		ηp^2^
	Mean ± SD [95% CI]	Mean ± SD [95% CI]	Mean ± SD [95% CI]				
Swim to approach	105.42 ± 21.81 [92.83–118.02]	104.28 ± 14.51 [95.90–112.66]	89.57 ± 9.86 [83.87–95.26]	0.001	R vs. R-LF:	0.003	0.401
					R vs. R-SF:	1.000	
					R-LF vs. R-SF	0.005	
Tow-in to the shore	206.35 ± 47.49 [178.93–233.77]	152.21 ± 32.46 [133.46–170.95]	130.57 ± 17.27 [120.60–140.57]	<0.001	R vs. R-LF:	<0.001	0.778
					R vs. R-SF:	<0.001	
					R-LF vs. R-SF	0.041	
Overall rescue time	311.78 ± 61.49 [276.28–347.28]	256.50 ± 41.60 [232.48–280.51]	220.14 ± 24.65 [205.90–234.38]	<0.001	R vs. R-LF:	<0.001	0.775
					R vs. R-SF:	<0.001	
					R-LF vs. R-SF	0.003	

R = rescue without fins; R-SF = rescue with short fins; R-LF: rescue with long fins; ηp^2^ = partial eta-squared.

**Table 2:** Physiological variables.

Physiological Variables	R	R-SF	R-LF	*p*-Value	Bonferroni Test		ηp^2^
	Mean ± SD [95% CI]	Mean ± SD [95% CI]	Mean ± SD [95% CI]				
RPE (Overall)	7.37 ± 1.35 [7.35–8.09]	6.78 ± 1.57 [6.78–7.69]	6.42 ± 1.50 [5.56–7.29]	0.047	R vs. R-LF:	0.045	0.209
					R vs. R-SF:	0.364	
					R-LF vs. R-SF	0.979	
RPE (Chest)	5.71 ± 1.93 [5.71–6.83]	5.28 ± 2.01 [5.28–6.45]	5.14 ± 12.03 [5.14–6.31]	0.526	R vs. R-LF:	0.841	0.048
					R vs. R-SF:	1.000	
					R-LF vs. R-SF	1.000	
RPE (Arms)	6.71 ± 2.55 [5.23–8.18]	5.00 ± 1.88 [3.91–6.08]	4.35 ± 1.90 [3.25–5.45]	< 0.001	R vs. R-LF:	0.010	0.445
					R vs. R-SF:	<0.001	
					R-LF vs. R-SF	0.717	
RPE (Legs)	6.92 ± 1.54 [6.03–7.81]	7.14 ± 2.07 [5.94–8.33]	7.35 ± 2.09 [6.14–8.56]	0.668	R vs. R-LF:	1.000	0.031
					R vs. R-SF:	1.000	
					R-LF vs. R-SF	1.000	
LACTATE Post rescue	7.29 ± 1.70 [6.30–8.27]	7.64 ± 1.80 [6.60–8.68]	7.95 ± 1.48 [7.09–8.80]	0.193	R vs. R-LF:	0.984	0.119
					R vs. R-SF:	0.218	
					R-LF vs. R-SF	1.000	

R = rescue without fins; R-SF = rescue with short fins; R-LF: rescue with long fins; ηp^2^ = partial eta-squared.

## Data Availability

The data presented in this study are available on request from the corresponding author. The data are not publicly available due to containing information protected by the Spanish Organic Law on Personal Data Protection and Digital Rights Guarantee.
